# Accounting for TLD response to CBCT protocols in external beam radiotherapy dose monitoring

**DOI:** 10.1002/acm2.70103

**Published:** 2025-04-24

**Authors:** Andrew J. White, Cliff G. Hammer, Matthew W. Brenner, Larry A. DeWerd, Kurt E. Stump, Wesley S. Culberson

**Affiliations:** ^1^ Department of Medical Physics School of Medicine and Public Health University of Wisconsin‐Madison Madison Wisconsin USA; ^2^ Department of Radiation Oncology Mercyhealth Javon Bea Hospital Rockford Illinois USA; ^3^ Department of Radiation Oncology Mission Cancer Center Asheville North Carolina USA

**Keywords:** CBCT, CIED, dose verification, in vivo dosimetry, TLD

## Abstract

**Purpose:**

LiF thermoluminescent dosimeters (TLDs) are commonly used in radiation therapy to verify the delivered dose. Examples include dose verification for complicated treatment setups or cardiovascular implantable electronic devices (CIEDs). TLDs may be present for both the pre‐treatment imaging kilovoltage (kV) beam and the megavoltage (MV) treatment beams. For low energy x‐ray beams, where the photoelectric effect dominates, TLDs respond differently than tissue or water. An overresponse of up to 40% has been previously reported for lower‐energy (kV) x‐rays when calibrated to higher‐energy (MV) beams. In this work, the response of TLDs to various cone beam computed tomography (CBCT) protocols with calibrations in clinical therapy beams (MV) is quantified.

**Methods:**

Three Varian OBI (Head, Thorax, and Pelvis) and three Elekta XVI (Fast Head and Neck, Fast Chest, and Fast Pelvis) CBCT protocols were investigated. For each protocol, TLDs were positioned on a tissue equivalent phantom at various distances extending out from the center of the imaging field. The response was determined by calibrating TLDs to a 6 MV photon beam.

**Results:**

The maximum in‐field TLD response ranged from 0.82 to 4.80 and 0.06 to 2.69 cGy for Varian OBI and Elekta XVI protocols, respectively. The out‐of‐field CBCT response dropped exponentially from the field edge. Calculated uncertainties were generally less than 3% (*k* = 1), with exceptions along the edge of the CBCT field (6%) and at the most distal TLD positions (34%).

**Conclusions:**

Using the measured TLD responses to CBCT protocols with an MV calibration, the therapeutic dose can be isolated. The therapeutic dose can then be compared to predictions from the treatment planning system (TPS), allowing for more accurate dose verification for complex treatment setups and patients with CIEDs. The CBCT response can change the reported therapeutic dose by up to 2.5%.

## 1 INTRODUCTION

Modern treatment planning systems (TPS) are becoming increasingly accurate at calculating the expected dose distribution for patients receiving external beam radiation therapy. However, one limitation is the lack of consideration of pre‐treatment imaging dose. Additionally, some treatments require complex setups that challenge the capabilities of the TPS to accurately determine the expected therapeutic dose. Examples of complex setups include the use of bolus to increase coverage near the surface, around skin folds, along abutting fields, and other unique geometries. In these situations, it is common to utilize in vivo dosimetry on the first fraction of treatment to verify the dose calculated by the TPS and make adjustments for future fractions if necessary.^1^

Many patients receiving radiation therapy have cardiovascular implantable electronic devices (CIEDs) to help manage existing heart conditions. The American Association of Physicists in Medicine (AAPM) Task Group 203 has provided an extensive framework on how to manage CIEDs through every step of the radiation oncology workflow. Due to their low radiation tolerance, CIEDs require additional considerations to minimize the risk of damage / malfunction during treatment. For example, treatment plans are produced that attempt to avoid primary transmission and reduce secondary transmission (scatter) through the device. However, due to their centrally located position on the body, the devices often come in close proximity to radiation fields. Common sites that impact CIEDs include lung, breast, and head and neck cases. In these instances, measurement of the cumulative dose received by the CIED is recommended. In vivo measurements are typically performed for the first fraction of treatment and compared to the dose per fraction estimated by the TPS.^2^

Thermoluminescent dosimeters (TLDs) are one of the few dosimeters that are commonly used for the in vivo measurements discussed above.^1^, ^2^, ^3^ Unfortunately, like many solid‐state dosimeters, TLDs have an associated energy dependence compared to water due to the high atomic number of the base material.^3^ Nunn et al. reported the response of TLD‐100 to be up to 37% higher at kV energies, relative to ^60^Co.^4^ This energy dependence complicates dose measurements for multimodal deliveries, such as when a CBCT is acquired prior to a treatment to verify patient alignment.

The dose contribution from pre‐treatment imaging procedures is commonly ignored, especially since imaging is not required to be performed before each fraction. However, when an in vivo dosimeter is in place for both the imaging procedure as well as the treatment, the dosimeter will absorb the dose from the imaging and treatment part of the procedure. It would be useful to be able to separate out the two components of the dose. This would help compare more accurately the predicted dose from the TPS with the therapeutic dose delivered. The biggest challenge is that the dosimeters used for in vivo dosimetry respond differently to the lower energy x‐rays from the imaging procedures. This presents a challenge that we are trying to address in this investigation.

Two clinical methods are commonly followed when placing TLDs on a patient to verify the first fraction dose. Each method has advantages and limitations. The first method is to place the TLDs on the patient after the CBCT has been acquired. This eliminates the challenges associated with a multimodal delivery but increases the chances of patient motion / misalignment because someone must enter the treatment vault to position the TLDs after the appropriate couch shifts have been applied to align the patient for treatment. The total dose for fractions that include imaging is determined using Equation 1:

(1) $$D_{\mathrm{CBCT}+\mathrm{ther}\mathrm{apeu}\mathrm{tic}}=D_{\mathrm{CBCT}}^{\mathrm{kVcal}}+\left(R_{\mathrm{ther}\mathrm{apeu}\mathrm{tic}}^{\mathrm{MVcal}}\times K_{\mathrm{NR}}\right)$$

where $D_{\mathrm{CBCT}+\mathrm{ther}\mathrm{apeu}\mathrm{tic}}$ is the total absorbed dose to water at the measurement position, $D_{CBCT}^{kV\;cal}$ is the CBCT imaging dose determined from a kV calibration, $R_{\mathrm{ther}\mathrm{apeu}\mathrm{tic}}^{\mathrm{MV}\;\mathrm{cal}}$ is the therapeutic TLD response determined from an MV calibration, and $K_{NR}$ is an energy correction factor that accounts for MV beam softening outside the treatment field. Scarboro et al. have published relevant values for $K_{NR}$, which is defined to be unity along the central axis of the MV beam.^5^ Previous studies have also provided values for $D_{CBCT}^{kV\;cal}$ from various vendors and protocols, using the kV dose calibration to account for the energy dependence of the dosimeters.^6^, ^7^, ^8^, ^9^, ^10^, ^11^, ^12^

The second method is to place the TLDs on the patient prior to the CBCT. Couch shifts can be made, and the patient is treated without an additional trip into the treatment vault. However, this method leads to a more complex dosimetry problem due to the energy dependence of the TLDs. For the second method, it is necessary to know the CBCT response with an MV dose calibration. Using the overresponding CBCT component measured in this work, the therapeutic dose can be isolated and determined via subtraction from the total measured response (CBCT + therapeutic), as seen in Equation 2:

(2) $$D_{\mathrm{ther}\mathrm{apeu}\mathrm{tic}}=\left(R_{\mathrm{CBCT}+\mathrm{ther}\mathrm{apeu}\mathrm{tic}}^{\mathrm{MVcal}}-R_{\mathrm{CBCT}}^{\mathrm{MVcal}}\right)\times K_{\mathrm{NR}}$$

where $D_{\mathrm{ther}\mathrm{apeu}\mathrm{tic}}$ is the therapeutic absorbed dose to water at the measurement point, $R_{\mathrm{CBCT}+\mathrm{ther}\mathrm{apeu}\mathrm{tic}}^{\mathrm{MV}\;\mathrm{cal}}$ is the combined CBCT and therapeutic TLD response determined from an MV calibration (such as what is provided on a report from a TLD service), $R_{CBCT}^{MV\;cal}$ is the CBCT TLD response determined from an MV calibration, and $K_{NR}$ is an energy correction factor that accounts for MV beam softening outside the treatment field. Note that Equation 2 provides the therapeutic dose without any contribution from imaging.

This work aims to report the TLD response $\left(R_{CBCT}^{MV\;cal}\right)$ for various imaging protocols on both the Varian OBI and Elekta XVI imaging systems. The results of this work can be applied to help verify first fraction dose calculated by the TPS for complex treatment setups and patients with CIEDs.

## 2 METHODS AND MATERIALS

### 2.1 TLD procedures

One hundred fifty Harshaw TLD‐100 chips (3.2 × 3.2 × 0.89 mm^3^) (Thermo Electron Corporation, Oakwood Village, OH) were sorted based on their sensitivity and reproducibility before experimental measurements were conducted. The TLDs were exposed at 2 m using a ^137^Cs irradiator and given an air kerma of 2.67 ± 0.03 cGy. To assess the reproducibility of each individual TLD, the coefficient of variation (COV) was computed across three distinct cycles of annealing, exposure, and readout. TLDs with a COV exceeding 1.5 percent were excluded from further analysis in this study.

TLDs that met the reproducibility criteria were assigned a chip factor (CF) that individually characterized the sensitivity of the dosimeter. CFs are determined by normalizing the average charge reading (from the three sorting cycles) of an individual TLD to the median charge reading of the entire group. CFs were applied to all subsequent measurements.

The Cameron method of annealing was used throughout this work.^13^ TLDs were heated to 400°C for 1 h, cooled on an aluminum slab for 30 min, then placed in an 80°C oven for 24 h. After an additional 24 h, the TLDs were ready for measurements.

TLDs were read out at least 24 h post‐irradiation using a Harshaw 5500 automatic gas reader. Fifty TLDs were loaded into a carousel. A vacuum pick raises each TLD into a stream of heated nitrogen gas. Starting from 50°C, the gas is heated at a constant rate of 15°C/s until it reaches a maximum temperature of 350°C. This maximum temperature is held until the total time of the time‐temperature profile (TTP) reaches 26.7 s. The TTP outlined above matches the clinical procedure of our commercial TLD service. The vacuum pick retracts, the carousel is rotated, and the process is repeated for all TLDs in the carousel.

During the TTP, emitted light is captured by an ET Enterprises Type 9125B24 photomultiplier tube (PMT). The area under the glow curve (integrated charge) was recorded and used during the analysis of this work.

Five unexposed TLDs were included in each readout to correct for background exposure. The average background reading was subtracted from the corrected charge readings of each individual TLD.

### 2.2 Dose calibration

A calibration curve was produced to convert the corrected charge readings (nC) to the quantity of absorbed dose to water (cGy). A Varian TrueBeam STx (Varian Medical System, Palo Alto, CA) was used to irradiate five TLDs to three known dose points (0.4, 5.0, and 30.0 cGy). The TLDs were irradiated by a 6X photon beam. The lower bound of the calibration curve was limited by the minimum MU delivery of the linac (1.0 MU). The TLDs were placed at a depth of 19.05 cm in Virtual Water (Standard Imaging, Middleton, WI) to help achieve doses applicable to imaging. To account for backscatter, 10 cm of Virtual Water was also placed below the TLDs. The absorbed dose to water at the calibration depth was verified by placing an ADCL‐calibrated Exradin A12 (Standard Imaging, Middleton, WI) ion chamber at the same depth and applying appropriate TG‐51 corrections.

A linear fit was applied to the calibration data, and the resulting fit was used to determine the response of TLDs irradiated with various CBCT protocols. It is important to note that the quantity of absorbed dose used in the calibration curve is only valid for the MV beam used during calibrations. Due to the overresponse of TLDs at lower x‐ray energies, the absorbed dose to water from the CBCT protocols is lower than what is determined when using the MV calibration curve. However, the aim of this study is to provide the information needed to remove the CBCT response from the rest of the megavoltage signal. To that end, a single MV calibration curve is used.

### 2.3 Experimental setup and TLD positioning

Multiple phantoms were combined to produce a setup that mimics the full patient anatomy: a Catphan phantom (Phantom Laboratory, Greenwich, NY) was used for the head, a Lungman phantom (Kyoto Kagaku, Kyoto, Japan) for the torso, and a custom pelvic phantom (Kyoto Kagaku, Kyoto, Japan) for the pelvis.

**FIGURE 1:**
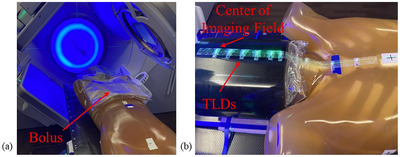
Photographs of (a) phantom setup with flexible bolus for head CBCT protocols and (b) TLD positioning on the phantom, with the leftmost placement in alignment with the center of the CBCT imaging field. CBCT, cone beam computed tomography; TLD, thermoluminescent dosimeter.

Nine sets of three TLDs were sealed in plastic and positioned at the following distances along the IEC negative *Y*‐axis: 0, 3, 6, 9, 12, 14, 20, 25, and 30 cm. The 0 cm position is the machine isocenter as determined by the wall lasers, representing the center of the imaging field. Three different phantom isocenters were used to mimic the anatomy of the three protocols investigated. Flexible bolus (2 cm) was placed over the TLDs for all measurements. The experimental setup is shown in Figure 1.

The TLD SNR from a single CBCT protocol was too low to achieve reproducible results. To increase the signal, multiple CBCTs were performed for each protocol. For the Varian protocols, the following number of scans were delivered: Head (10), Thorax (7), and Pelvis (3). For the Elekta protocols, the following number of scans were delivered: Fast Head and Neck (15), Fast Chest (7), and Fast Pelvis (3). The number of scans for each protocol was selected to balance the need for a higher SNR and the limitations of the anode heating capacity.

### 2.4 CBCT protocols

#### 2.4.1 Varian OBI

A Varian TrueBeam STx was used to investigate three OBI CBCT protocols during this work (Head, Thorax, and Pelvis). The selected protocols represent scans that are commonly used in the clinic. The CBCT parameters for the Varian OBI protocols are outlined in Table 1.

**TABLE 1:** Default scan parameters for Varian OBI CBCT protocols.

	Varian CBCT Protocol		
Parameter	Head	Thorax	Pelvis
**Voltage (kV)**	100	125	125
**Current (mA)**	15	15	60
**Exposure time (ms)**	20	20	20
**Fan type**	Full	Half	Half
**Number of projections**	900	900	900
**Trajectory**	Full	Full	Full
**Exposure (mAs)**	270	270	1080

*Note*: These parameters can be adjusted by the user.

Abbreviation: CBCT, cone beam computed tomography.

#### 2.4.2 Elekta XVI

An Elekta Versa HD (Elekta, Stockholm, Sweden) was used to investigate three XVI CBCT protocols during this work (Fast Head (S20), Fast Chest (M20), and Fast Pelvis (M20)). Elekta XVI systems have additional variables, such as filtration and field‐of‐view, that can be manually changed by the user for each protocol. Parameters similar to those used in clinical workflows were chosen. The CBCT parameters for the Elekta XVI protocols are outlined in Table 2.

**TABLE 2:** Default scan parameters for Elekta XVI CBCT protocols.

	Elekta CBCT Protocol		
Parameter	Fast head and neck (S20)	Fast chest (M20)	Fast pelvis (M20)
**Voltage (kV)**	100	120	120
**kV Collimator**	S20	M20	M20
**kV Filter**	F0	F1	F1
**Number of projections**	183	330	330
**Trajectory**	Half	Full	Full
**Start/Stop angle (°)**	160–320	180–180	180–180
**Exposure (mAs)**	18.3	132	528

*Note*: These parameters can be adjusted by the user.

Abbreviation: CBCT, cone beam computed tomography.

## 3 RESULTS

To match clinical use, this study used a MV calibration curve in which the TLD overresponse from the CBCT was intentionally left unaccounted for. It is acknowledged that this method results in a value (termed “response” throughout this work) that is not representative of the physical absorbed dose to water, nor the absorbed dose to the TLDs. The reason for reporting the results this way was to provide the values needed to adjust the final reported TLD results provided by in vivo TLD dosimetry services, where a single MV calibration curve is used. Reported TLD response from the CBCT can be subtracted from the total response provided in the report, allowing for an isolated therapeutic dose per fraction.

The measured responses from the Varian OBI protocols were higher than the Elekta XVI protocols for each site investigated, which was expected due to the larger number of projections used in the acquisitions (900 vs. 183/330), leading to higher mAs. Additionally, the Varian OBI protocols utilize a slightly higher tube voltage in the Chest and Pelvis scans (125 vs. 120 kV).

All uncertainties are provided at one standard deviation of the mean (*k* = 1 confidence level). Lookup tables are provided in the Appendix (Table A1—Varian OBI and Table A2—Elekta XVI) to conveniently reference values at specific measurement points.

### 3.1 Varian OBI

**FIGURE 2:**
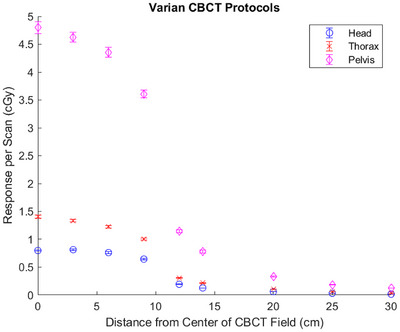
Measured response per scan as a function of distance from the center of the CBCT field for all Varian OBI CBCT protocols investigated. Error bars represent *k* = 1 uncertainties. CBCT, cone beam computed tomography.

The measured responses for each Varian OBI protocol are plotted in Figure 2. The maximum response from the Head, Thorax, and Pelvis protocols were 0.82 ± 0.01, 1.41 ± 0.03, and 4.80 ± 0.11 cGy, respectively. At the most distal measurement point (30 cm), the responses were 0.017 ± 0.001, 0.035 ± 0.001, and 0.124 ± 0.002 cGy, respectively.

### 3.2 Elekta XVI

**FIGURE 3:**
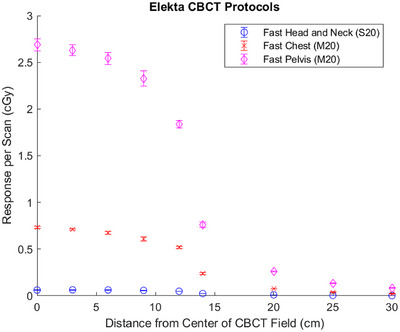
Measured response per scan as a function of distance from the center of the CBCT field for all Elekta XVI CBCT protocols investigated. Error bars represent *k* = 1 uncertainties. CBCT, cone beam computed tomography.

Similarly, the measured responses for each Elekta XVI protocol are plotted in Figure 3. The maximum responses from the Fast Head and Neck, Fast Chest, and Fast Pelvis protocols were 0.063 ± 0.003, 0.73 ± 0.01, and 2.69 ± 0.06 cGy, respectively. At the most distal measurement point (30 cm), the responses were 0.002 ± 0.001, 0.022 ± 0.001, and 0.082 ± 0.003 cGy, respectively.

## 4 DISCUSSION

This work provides clinical physicists with data needed to accurately interpret TLD in vivo measurements when a CBCT is acquired before treatment. By reporting the CBCT response with an MV dose calibration, the results can be subtracted from the total response provided on the in vivo TLD report. With the isolated imaging and therapeutic doses known, clinical physicists have more flexibility in accurately determining the dose at the point of interest over the course of the entire treatment, regardless of imaging frequency. Additionally, this data will allow clinics to place TLDs prior to the CBCT, reducing setup errors caused by patient motion after couch shifts have been applied.

In this work, CBCT response was measured inside and outside of the imaging field. The results demonstrate that out‐of‐field CBCT response drops exponentially from the field edge. Similar results have been reported in other previous studies.^14^, ^15^, ^16^, ^17^

The results support that in‐field CBCT response is substantial and should be considered for treatments that include many fractions with imaging. Perks et al. reported that the in‐field dose from a kV CBCT was comparable to the out‐of‐field intensity modulated radiation therapy (IMRT) dose.^14^ Based on the results of this work, failure to account for the CBCT response can lead to total dose verification errors of up to 2.5% (assuming a therapeutic dose of 180 cGy/fx) when TLDs are placed in the imaging field prior to treatment.

When using the results presented in this work to make corrections to in vivo TLD measurements, one must also consider the out‐of‐field spectral changes and associated TLD overresponse to the scattered (MV) treatment beam. Scarboro et al. have provided a TLD energy correction factor for MV beams as a function of the mean photon energy.^5^ This correction factor should be applied after the CBCT response has been subtracted out (see Equation 2), as it only applies to the MV beam contribution, which was not measured in this work. In the most extreme cases, the magnitude of this correction can be up to 12% depending on field size and depth of measurement.

Both energy corrections outlined above (the CBCT response reported in this work and the out‐of‐field MV softening reported by Scarboro et al.) result in TLD readings higher than the true absorbed dose to water at the measurement point. This work has provided the data to more accurately determine the true absorbed dose to water for TLD dose verifications. Not accounting for the energy dependence of the TLDs can lead to overly conservative dose estimates.

### 4.1 CIED dose monitoring

CIED dose tolerances have been studied by many groups in the past and are discussed extensively in TG‐203. Recommendations of the task group include in vivo monitoring of devices within 10 cm of the treatment field, keeping the cumulative dose to the device under 2 Gy, and avoiding energies > 10 MV due to neutron production.^2^ Without appropriate precautions throughout the radiation therapy workflow, devices can malfunction during treatment, putting the safety of the patient in danger. However, by using in vivo dosimetry to verify that the dose to the device is below the manufacturer's recommendation, the risk of complications is significantly reduced.^2^ Using the data from this work, the dose to cardiac devices can be tracked more accurately.

### 4.2 Heel effect

The self‐attenuation of photons on the anode side of the x‐ray tube causes the heel effect to be observed in the measurements. However, the orientation of the x‐ray tube for the two vendors investigated in this work is not the same. The Varian OBI system orients the x‐ray tube along the IEC *Y‐*axis, while the Elekta XVI tube is positioned orthogonal to the IEC *Y*‐axis.

**FIGURE 4:**
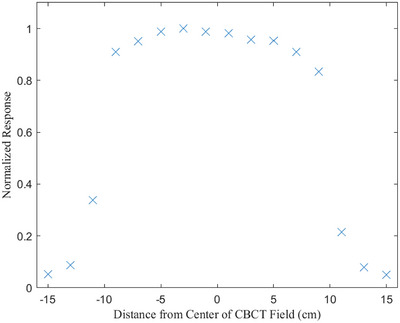
Normalized response plotted as a function of distance from the center of the fields from a Varian OBI scan. Each potted data point represents the average of three TLD measurements. Note the observable heel effect. TLD, thermoluminescent dosimeter.

Because the TLDs were positioned down the midline of the phantom, along the superior–inferior direction of the phantom, only the measurements of the Varian OBI protocols were impacted by the heel effect. Figure 4 shows a full profile for a Varian OBI protocol. The negative x values correspond to the cathode side of the x‐ray tube (closest to the gantry). The measured signal difference between the anode and cathode sides of the profile was less than 5%. Yuasa et al. reported similar impacts of the heel effect for Varian OBI CBCT protocols.^18^

Similarly, Marchant and Joshi observed the heel effect when taking dose profiles of Elekta XVI protocols.^19^ The dosimetric impact of the heel effect is more complex for the Elekta XVI system due to the orientation of the x‐ray tube being orthogonal to the gantry's axis of rotation. Although only placing TLDs down the midline of the phantom simplified the presentation of the results, it does not consider off‐axis effects. CBCT protocols that do not complete a full revolution of the gantry (such as the XVI Fast Head and Neck protocol investigated in this work) require further considerations based on the start and stop angles of the gantry in relation to the placement of the TLDs. Such considerations extend beyond the scope of this work, as one of the main applications was for CIEDs—where dose verifications are typically only necessary on the anterior side of the patient. Giaddui et al. have published on the angular dependence of CBCT dose for OBI and XVI protocols using OSLDs and film.^6^

### 4.3 Patient size

Because CBCT systems revolve around the patient, dose is deposited from all gantry angles. For this reason, it is not sufficient to exclusively consider the entrance dose on the dosimeter—exit dose is also recorded. Because patient size is directly related to the attenuation of the x‐rays and scatter, it must be considered if it deviates significantly from a standard‐sized patient. The phantom used for all measurements was representative of a standard‐sized patient.

### 4.4 Uncertainty

A standard uncertainty table is provided in Table 3. This table is representative of measurement points within the CBCT field. The uncertainties at the edge of the profile, as well as far outside the field, were higher for two reasons: the TLD positioning and the Type A statistical uncertainty caused by low SNR. Because these two uncertainty components are functions of the radial distance from the center of the imaging field, they were individually calculated for each measurement position. The individually calculated uncertainties are included in Figures 2 and 3, as well as Tables A1 and A2.

**TABLE 3:** Standard uncertainty table for TLDs used in this work.

Parameter	Type A	Type B	
TLD reproducibility	1.50		
Dose determination		0.90	
TLD positioning		0.20	
PMT linearity		0.30	
Reader stability		0.20	
Quadratic sum	1.50	0.99	
A and B quadratic sum	1.80		
Measured TLD uncertainty		1.80	*k* = 1
		3.59	*k* = 2

*Note*: Individual uncertainties were determined for each scan type/radial distance by individually considering the differences in Type A statistical uncertainty and Type B positional uncertainty.

Abbreviation: TLD, thermoluminescent dosimeter.

At the edges of the profile, where the gradient is steeper, the uncertainty is higher. In this region, a small change in TLD positioning leads to a large change in measured signal. To account for the change in uncertainty as a function of radial distance, a fit was applied to the profile and shifted 1 mm in either direction. The maximum percent signal deviation at each measurement point was used as the TLD positioning uncertainty. In the steepest portion of the profile, a 1 mm shift changed the response by up to 4.8%.

**FIGURE 5:**
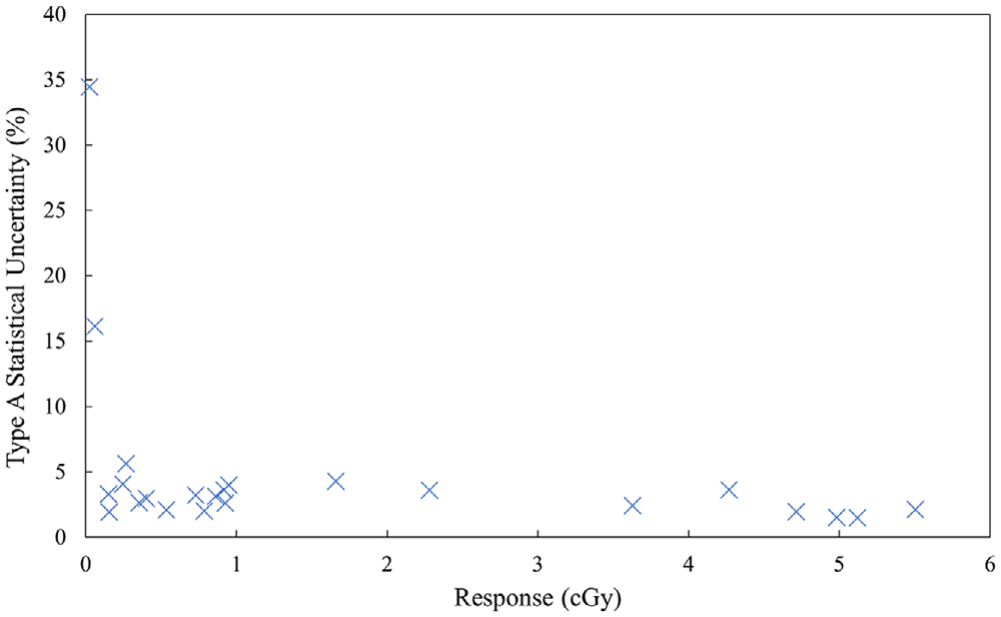
Type A statistical uncertainty as a function of measured response. The reproducibility of the TLD measurements decreased dramatically below 0.1 cGy. TLD, thermoluminescent dosimeter.

At the most distal edges of the profile (outside the CBCT field), the measured thermoluminescence was low, leading to noisy glow curves. The decrease in SNR led to higher Type A statistical uncertainties between the three TLDs at these measurement positions. The low signal was more prominent with the Elekta protocols, as they were measured using the “fast” acquisition, which utilizes half the standard number of projections. The Type A statistical uncertainty for the Elekta XVI Fast Head and Neck protocol was 34.5% at 30 cm. At this measurement point, the total response from the 15 scans was less than 0.03 cGy—below what was found to yield reproducible TLD readouts. The Type A statistical uncertainty as a function of response is shown in Figure 5. As seen in the figure, signal less than 0.1 cGy resulted in high statistical uncertainty during our measurements. The total signal was increased by repeating each CBCT protocol multiple times but was limited by the heating capacity of the x‐ray anode.

## 5 CONCLUSION

TLDs are common in vivo dosimeters that are used during the first fraction of some treatments to verify the therapeutic dose calculated by the TPS. Examples include patients that require complex setups or have a CIED. To minimize dosimetric errors caused by patient motion between the CBCT and treatment delivery, the TLDs are often placed on the patient prior to imaging. In this case, the TLD readout represents the combined imaging and therapeutic responses. Due to the energy dependence of TLDs, an MV dose calibration will cause an overresponse to the kV CBCT protocol. This work provides the TLD response (with an MV dose calibration) for multiple Varian OBI and Elekta XVI CBCT protocols. The provided CBCT TLD response can be subtracted from the total response reported by in vivo TLD services to more accurately isolate and verify the therapeutic dose estimated by the TPS.

## AUTHOR CONTRIBUTION

Wesley Culberson contributed to the research idea and supervised the project. Kurt Stump contributed to the research idea. Cliff Hammer aided in the project design and data analysis. Matthew Brenner helped perform measurements on the Elekta XVI system. Larry DeWerd contributed to the research idea. Andrew White performed measurements, performed the scientific analysis, and wrote the manuscript. All authors discussed the results and edited the final manuscript.

## CONFLICT OF INTEREST STATEMENT

The authors declare no conflicts of interest.

## 1

**TABLE A1:** Measured response per scan as a function of distance from the center of the CBCT field for Varian OBI protocols.

Distance from center of imaging field (cm)	Measured response from Varian OBI CBCT protocols (cGy)		
	Head	Thorax	Pelvis
**0**	0.80 ± 0.01	1.41 ± 0.03	4.80 ± 0.11
**3**	0.82 ± 0.01	1.33 ± 0.02	4.62 ± 0.09
**6**	0.76 ± 0.02	1.23 ± 0.03	4.35 ± 0.09
**9**	0.65 ± 0.01	1.01 ± 0.02	3.61 ± 0.07
**12**	0.193 ± 0.005	0.31 ± 0.01	1.14 ± 0.03
**14**	0.128 ± 0.003	0.217 ± 0.005	0.78 ± 0.03
**20**	0.061 ± 0.001	0.107 ± 0.002	0.33 ± 0.01
**25**	0.032 ± 0.001	0.058 ± 0.002	0.183 ± 0.004
**30**	0.017 ± 0.001	0.035 ± 0.001	0.124 ± 0.002

*Note*: Uncertainties are reported at *k* = 1.

Abbreviation: CBCT, cone beam computed tomography.

**TABLE A2:** Measured response per scan as a function of distance from the center of the CBCT field for Elekta XVI protocols.

	Measured response from Elekta XVI CBCT protocols (cGy)		
Distance from center of imaging field (cm)	Fast head and neck	Fast chest	Fast pelvis
**0**	0.061 ± 0.002	0.73 ± 0.01	2.69 ± 0.06
**3**	0.063 ± 0.003	0.71 ± 0.01	2.63 ± 0.06
**6**	0.062 ± 0.002	0.67 ± 0.01	2.54 ± 0.06
**9**	0.057 ± 0.002	0.61 ± 0.02	2.33 ± 0.08
**12**	0.048 ± 0.002	0.52 ± 0.01	1.84 ± 0.04
**14**	0.024 ± 0.001	0.24 ± 0.01	0.76 ± 0.03
**20**	0.010 ± 0.001	0.077 ± 0.002	0.26 ± 0.01
**25**	0.004 ± 0.001	0.038 ± 0.002	0.134 ± 0.004
**30**	0.002 ± 0.001	0.022 ± 0.001	0.082 ± 0.003

*Note*: Uncertainties are reported at *k* = 1.

Abbreviation: CBCT, cone beam computed tomography.
